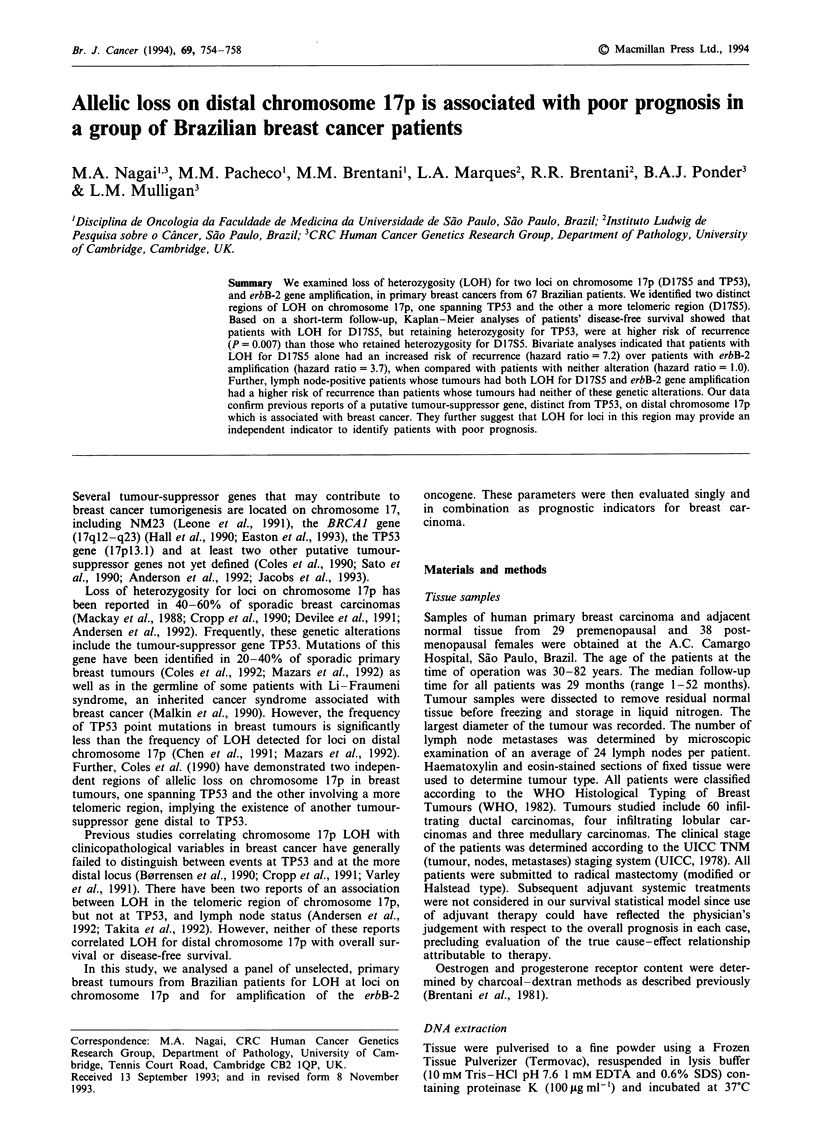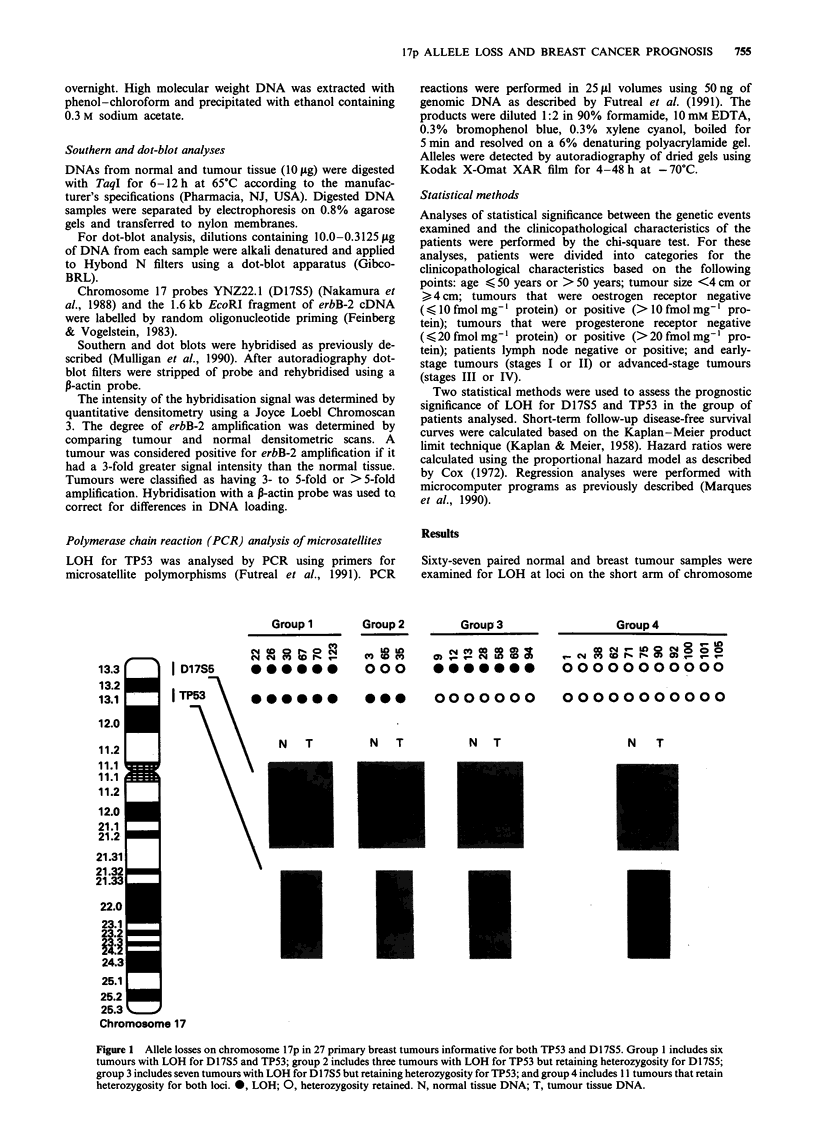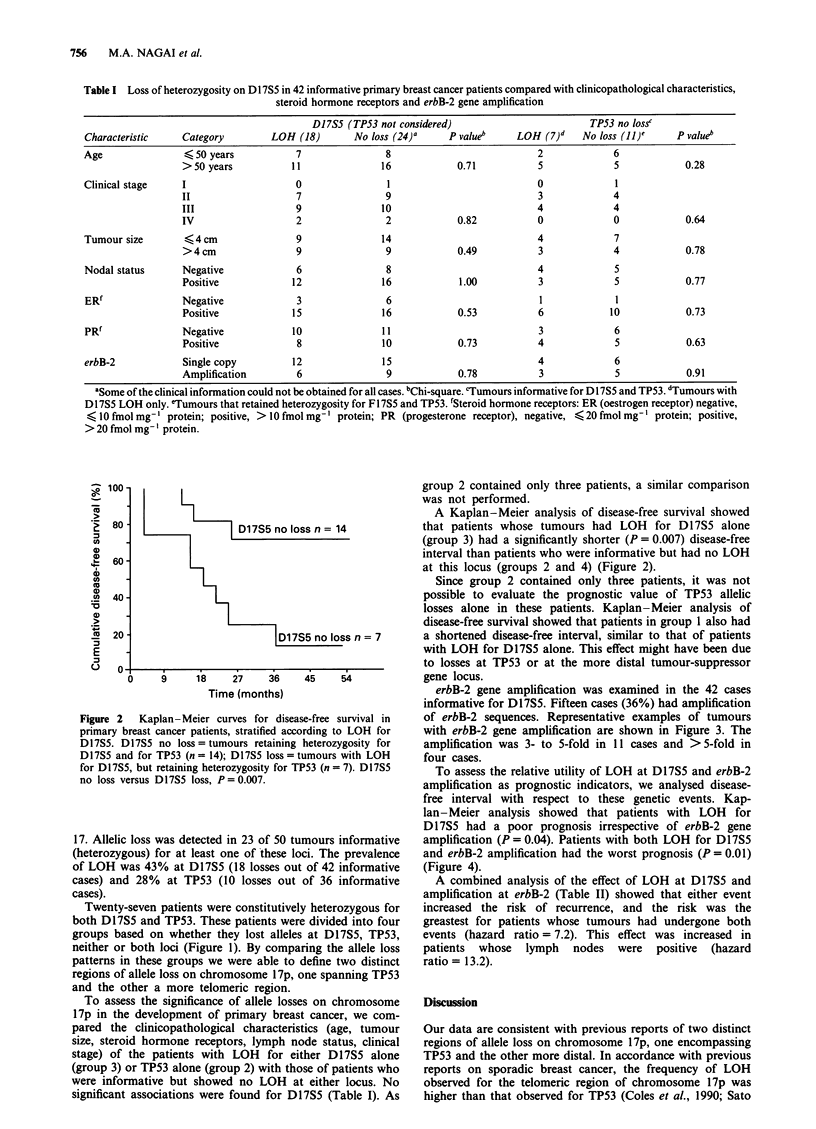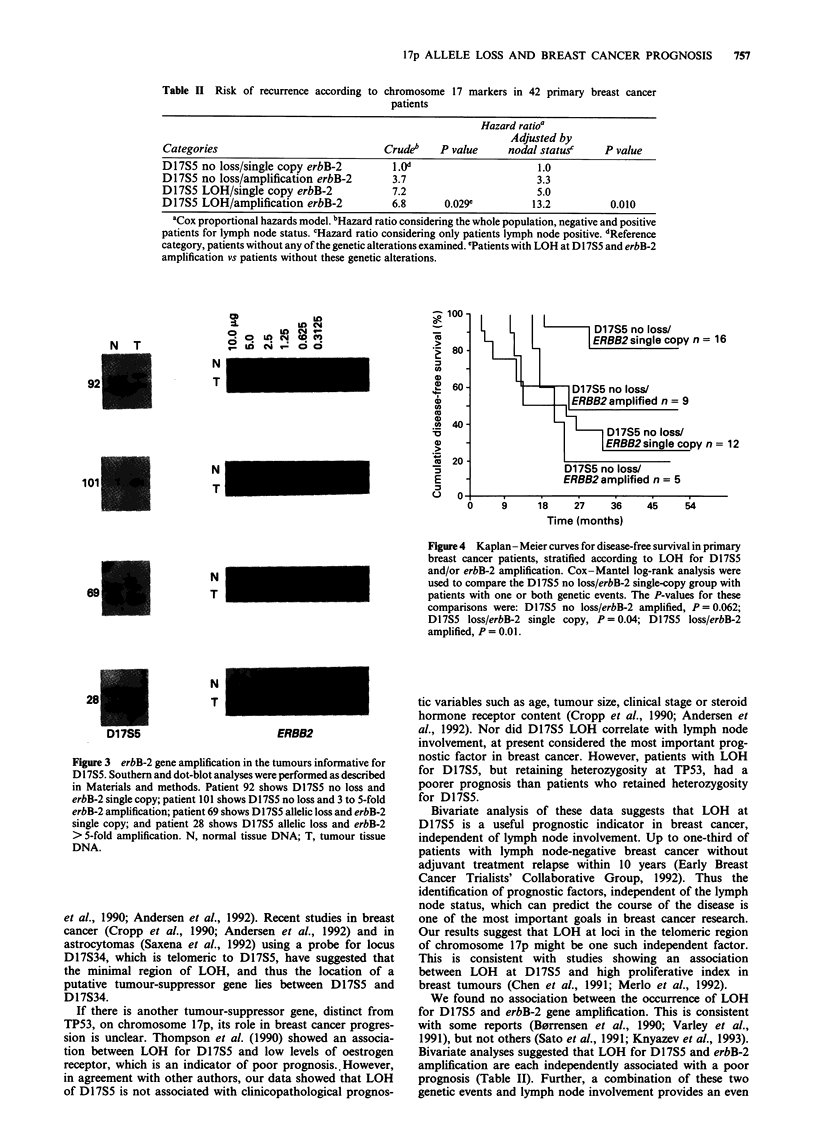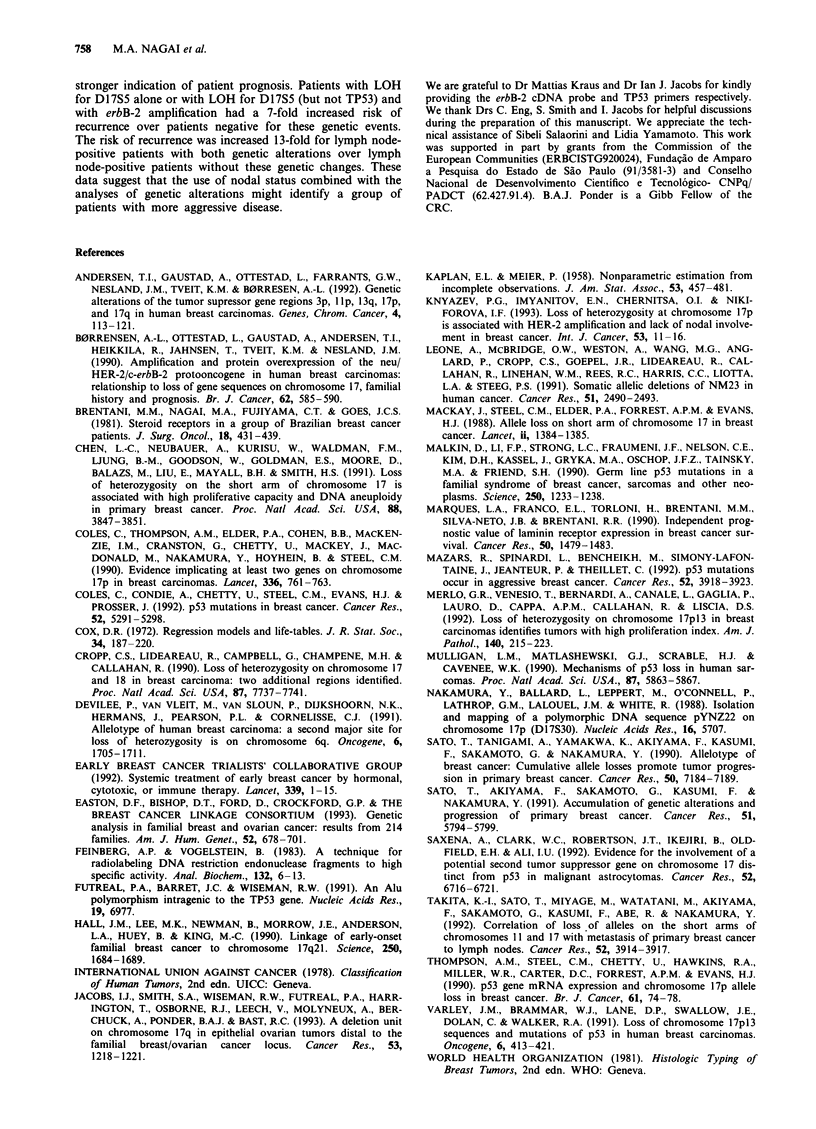# Allelic loss on distal chromosome 17p is associated with poor prognosis in a group of Brazilian breast cancer patients.

**DOI:** 10.1038/bjc.1994.142

**Published:** 1994-04

**Authors:** M. A. Nagai, M. M. Pacheco, M. M. Brentani, L. A. Marques, R. R. Brentani, B. A. Ponder, L. M. Mulligan

**Affiliations:** Faculdade de Medicina, Universidade de São Paulo, Brazil.

## Abstract

**Images:**


 © Macmillan Press Ltd., 1994

### Allelic loss on distal chromosome 17p is associated with poor prognosis in a group of Brazilian breast cancer patients

M.A. Nagai[1,3], M.M. Pacheco[1], M.M. Brentani[1], L.A. Marques[2], R.R. Brentani[2], B.A.J. Ponder[3] & L.M. Mulligan[3]

[1]*Disciplina de Oncologia da Faculdade de Medicina da Universidade de São Paulo, São Paulo, Brazil;* [2]*Instituto Ludwig de Pesquisa sobre o Câncer, São Paulo, Brazil;* [3]*CRC Human Cancer Genetics Research Group, Department of Pathology, University of Cambridge, Cambridge, UK.*

**Summary** We examined loss of heterozygosity (LOH) for two loci on chromosome 17p (D17S5 and TP53), and *erb*B-2 gene amplification, in primary breast cancers from 67 Brazilian patients. We identified two distinct regions of LOH on chromosome 17p, one spanning TP53 and the other a more telomeric region (D17S5). Based on a short-term follow-up, Kaplan-Meier analyses of patients' disease-free survival showed that patients with LOH for D17S5, but retaining heterozygosity for TP53, were at higher risk of recurrence ($P = 0.007$) than those who retained heterozygosity for D17S5. Bivariate analyses indicated that patients with LOH for D17S5 alone had an increased risk of recurrence (hazard ratio = 7.2) over patients with *erb*B-2 amplification (hazard ratio = 3.7), when compared with patients with neither alteration (hazard ratio = 1.0). Further, lymph node-positive patients whose tumours had both LOH for D17S5 and *erb*B-2 gene amplification had a higher risk of recurrence than patients whose tumours had neither of these genetic alterations. Our data confirm previous reports of a putative tumour-suppressor gene, distinct from TP53, on distal chromosome 17p which is associated with breast cancer. They further suggest that LOH for loci in this region may provide an independent indicator to identify patients with poor prognosis.

Several tumour-suppressor genes that may contribute to breast cancer tumorigenesis are located on chromosome 17, including NM23 (Leone *et al.*, 1991), the *BRCA1* gene (17q12-q23) (Hall *et al.*, 1990; Easton *et al.*, 1993), the TP53 gene (17p13.1) and at least two other putative tumour-suppressor genes not yet defined (Coles *et al.*, 1990; Sato *et al.*, 1990; Anderson *et al.*, 1992; Jacobs *et al.*, 1993).

Loss of heterozygosity for loci on chromosome 17p has been reported in 40-60% of sporadic breast carcinomas (Mackay *et al.*, 1988; Cropp *et al.*, 1990; Devilee *et al.*, 1991; Andersen *et al.*, 1992). Frequently, these genetic alterations include the tumour-suppressor gene TP53. Mutations of this gene have been identified in 20-40% of sporadic primary breast tumours (Coles *et al.*, 1992; Mazars *et al.*, 1992) as well as in the germline of some patients with Li-Fraumeni syndrome, an inherited cancer syndrome associated with breast cancer (Malkin *et al.*, 1990). However, the frequency of TP53 point mutations in breast tumours is significantly less than the frequency of LOH detected for loci on distal chromosome 17p (Chen *et al.*, 1991; Mazars *et al.*, 1992). Further, Coles *et al.* (1990) have demonstrated two independent regions of allelic loss on chromosome 17p in breast tumours, one spanning TP53 and the other involving a more telomeric region, implying the existence of another tumour-suppressor gene distal to TP53.

Previous studies correlating chromosome 17p LOH with clinicopathological variables in breast cancer have generally failed to distinguish between events at TP53 and at the more distal locus (Børrensen *et al.*, 1990; Cropp *et al.*, 1991; Varley *et al.*, 1991). There have been two reports of an association between LOH in the telomeric region of chromosome 17p, but not at TP53, and lymph node status (Andersen *et al.*, 1992; Takita *et al.*, 1992). However, neither of these reports correlated LOH for distal chromosome 17p with overall survival or disease-free survival.

In this study, we analysed a panel of unselected, primary breast tumours from Brazilian patients for LOH at loci on chromosome 17p and for amplification of the *erb*B-2 oncogene. These parameters were then evaluated singly and in combination as prognostic indicators for breast carcinoma.

Correspondence: M.A. Nagai, CRC Human Cancer Genetics Research Group, Department of Pathology, University of Cambridge, Tennis Court Road, Cambridge CB2 1QP, UK.
Received 13 September 1993; and in revised form 8 November 1993.

### Materials and methods

### *Tissue samples*

Samples of human primary breast carcinoma and adjacent normal tissue from 29 premenopausal and 38 postmenopausal females were obtained at the A.C. Camargo Hospital, São Paulo, Brazil. The age of the patients at the time of operation was 30-82 years. The median follow-up time for all patients was 29 months (range 1-52 months). Tumour samples were dissected to remove residual normal tissue before freezing and storage in liquid nitrogen. The largest diameter of the tumour was recorded. The number of lymph node metastases was determined by microscopic examination of an average of 24 lymph nodes per patient. Haematoxylin and eosin-stained sections of fixed tissue were used to determine tumour type. All patients were classified according to the WHO Histological Typing of Breast Tumours (WHO, 1982). Tumours studied include 60 infiltrating ductal carcinomas, four infiltrating lobular carcinomas and three medullary carcinomas. The clinical stage of the patients was determined according to the UICC TNM (tumour, nodes, metastases) staging system (UICC, 1978). All patients were submitted to radical mastectomy (modified or Halstead type). Subsequent adjuvant systemic treatments were not considered in our survival statistical model since use of adjuvant therapy could have reflected the physician's judgement with respect to the overall prognosis in each case, precluding evaluation of the true cause-effect relationship attributable to therapy.

Oestrogen and progesterone receptor content were determined by charcoal-dextran methods as described previously (Brentani *et al.*, 1981).

### *DNA extraction*

Tissue were pulverised to a fine powder using a Frozen Tissue Pulverizer (Termovac), resuspended in lysis buffer (10 mM Tris-HCl pH 7.6 1 mM EDTA and 0.6% SDS) containing proteinase K ($100\,\mu g\,ml^{-1}$) and incubated at 37°C

overnight. High molecular weight DNA was extracted with phenol–chloroform and precipitated with ethanol containing 0.3 M sodium acetate.

*Southern and dot-blot analyses*

DNAs from normal and tumour tissue (10 µg) were digested with *Taq*I for 6–12 h at 65°C according to the manufacturer's specifications (Pharmacia, NJ, USA). Digested DNA samples were separated by electrophoresis on 0.8% agarose gels and transferred to nylon membranes.

For dot-blot analysis, dilutions containing 10.0–0.3125 µg of DNA from each sample were alkali denatured and applied to Hybond N filters using a dot-blot apparatus (Gibco-BRL).

Chromosome 17 probes YNZ22.1 (D17S5) (Nakamura *et al.*, 1988) and the 1.6 kb *Eco*RI fragment of *erb*B-2 cDNA were labelled by random oligonucleotide priming (Feinberg & Vogelstein, 1983).

Southern and dot blots were hybridised as previously described (Mulligan *et al.*, 1990). After autoradiography dot-blot filters were stripped of probe and rehybridised using a β-actin probe.

The intensity of the hybridisation signal was determined by quantitative densitometry using a Joyce Loebl Chromoscan 3. The degree of *erb*B-2 amplification was determined by comparing tumour and normal densitometric scans. A tumour was considered positive for *erb*B-2 amplification if it had a 3-fold greater signal intensity than the normal tissue. Tumours were classified as having 3- to 5-fold or >5-fold amplification. Hybridisation with a β-actin probe was used to correct for differences in DNA loading.

*Polymerase chain reaction (PCR) analysis of microsatellites*

LOH for TP53 was analysed by PCR using primers for microsatellite polymorphisms (Futreal *et al.*, 1991). PCR reactions were performed in 25 µl volumes using 50 ng of genomic DNA as described by Futreal *et al.* (1991). The products were diluted 1:2 in 90% formamide, 10 mM EDTA, 0.3% bromophenol blue, 0.3% xylene cyanol, boiled for 5 min and resolved on a 6% denaturing polyacrylamide gel. Alleles were detected by autoradiography of dried gels using Kodak X-Omat XAR film for 4–48 h at −70°C.

*Statistical methods*

Analyses of statistical significance between the genetic events examined and the clinicopathological characteristics of the patients were performed by the chi-square test. For these analyses, patients were divided into categories for the clinicopathological characteristics based on the following points: age ⩽50 years or >50 years; tumour size <4 cm or ⩾4 cm; tumours that were oestrogen receptor negative (⩽10 fmol mg$^{-1}$ protein) or positive (>10 fmol mg$^{-1}$ protein); tumours that were progesterone receptor negative (⩽20 fmol mg$^{-1}$ protein) or positive (>20 fmol mg$^{-1}$ protein); patients lymph node negative or positive; and early-stage tumours (stages I or II) or advanced-stage tumours (stages III or IV).

Two statistical methods were used to assess the prognostic significance of LOH for D17S5 and TP53 in the group of patients analysed. Short-term follow-up disease-free survival curves were calculated based on the Kaplan–Meier product limit technique (Kaplan & Meier, 1958). Hazard ratios were calculated using the proportional hazard model as described by Cox (1972). Regression analyses were performed with microcomputer programs as previously described (Marques *et al.*, 1990).

### Results

Sixty-seven paired normal and breast tumour samples were examined for LOH at loci on the short arm of chromosome

**Figure 1** Allele losses on chromosome 17p in 27 primary breast tumours informative for both TP53 and D17S5. Group 1 includes six tumours with LOH for D17S5 and TP53; group 2 includes three tumours with LOH for TP53 but retaining heterozygosity for D17S5; group 3 includes seven tumours with LOH for D17S5 but retaining heterozygosity for TP53; and group 4 includes 11 tumours that retain heterozygosity for both loci. ●, LOH; ○, heterozygosity retained. N, normal tissue DNA; T, tumour tissue DNA.

**Table I** Loss of heterozygosity on D17S5 in 42 informative primary breast cancer patients compared with clinicopathological characteristics, steroid hormone receptors and *erb*B-2 gene amplification

| | | *D17S5 (TP53 not considered)* | | | | *TP53 no loss*[c] | |
|---|---|---|---|---|---|---|---|
| *Characteristic* | *Category* | *LOH (18)* | *No loss (24)*[a] | *P value*[b] | *LOH (7)*[d] | *No loss (11)*[e] | *P value*[b] |
| Age | ≤50 years | 7 | 8 | | 2 | 6 | |
| | >50 years | 11 | 16 | 0.71 | 5 | 5 | 0.28 |
| Clinical stage | I | 0 | 1 | | 0 | 1 | |
| | II | 7 | 9 | | 3 | 4 | |
| | III | 9 | 10 | | 4 | 4 | |
| | IV | 2 | 2 | 0.82 | 0 | 0 | 0.64 |
| Tumour size | ≤4 cm | 9 | 14 | | 4 | 7 | |
| | >4 cm | 9 | 9 | 0.49 | 3 | 4 | 0.78 |
| Nodal status | Negative | 6 | 8 | | 4 | 5 | |
| | Positive | 12 | 16 | 1.00 | 3 | 5 | 0.77 |
| ER[f] | Negative | 3 | 6 | | 1 | 1 | |
| | Positive | 15 | 16 | 0.53 | 6 | 10 | 0.73 |
| PR[f] | Negative | 10 | 11 | | 3 | 6 | |
| | Positive | 8 | 10 | 0.73 | 4 | 5 | 0.63 |
| *erb*B-2 | Single copy | 12 | 15 | | 4 | 6 | |
| | Amplification | 6 | 9 | 0.78 | 3 | 5 | 0.91 |

[a]Some of the clinical information could not be obtained for all cases. [b]Chi-square. [c]Tumours informative for D17S5 and TP53. [d]Tumours with D17S5 LOH only. [e]Tumours that retained heterozygosity for F17S5 and TP53. [f]Steroid hormone receptors: ER (oestrogen receptor) negative, ≤10 fmol $mg^{-1}$ protein; positive, >10 fmol $mg^{-1}$ protein; PR (progesterone receptor), negative, ≤20 fmol $mg^{-1}$ protein; positive, >20 fmol $mg^{-1}$ protein.

**Figure 2** Kaplan–Meier curves for disease-free survival in primary breast cancer patients, stratified according to LOH for D17S5. D17S5 no loss = tumours retaining heterozygosity for D17S5 and for TP53 ($n = 14$); D17S5 loss = tumours with LOH for D17S5, but retaining heterozygosity for TP53 ($n = 7$). D17S5 no loss versus D17S5 loss, $P = 0.007$.

17. Allelic loss was detected in 23 of 50 tumours informative (heterozygous) for at least one of these loci. The prevalence of LOH was 43% at D17S5 (18 losses out of 42 informative cases) and 28% at TP53 (10 losses out of 36 informative cases).

Twenty-seven patients were constitutively heterozygous for both D17S5 and TP53. These patients were divided into four groups based on whether they lost alleles at D17S5, TP53, neither or both loci (Figure 1). By comparing the allele loss patterns in these groups we were able to define two distinct regions of allele loss on chromosome 17p, one spanning TP53 and the other a more telomeric region.

To assess the significance of allele losses on chromosome 17p in the development of primary breast cancer, we compared the clinicopathological characteristics (age, tumour size, steroid hormone receptors, lymph node status, clinical stage) of the patients with LOH for either D17S5 alone (group 3) or TP53 alone (group 2) with those of patients who were informative but showed no LOH at either locus. No significant associations were found for D17S5 (Table I). As group 2 contained only three patients, a similar comparison was not performed.

A Kaplan–Meier analysis of disease-free survival showed that patients whose tumours had LOH for D17S5 alone (group 3) had a significantly shorter ($P = 0.007$) disease-free interval than patients who were informative but had no LOH at this locus (groups 2 and 4) (Figure 2).

Since group 2 contained only three patients, it was not possible to evaluate the prognostic value of TP53 allelic losses alone in these patients. Kaplan–Meier analysis of disease-free survival showed that patients in group 1 also had a shortened disease-free interval, similar to that of patients with LOH for D17S5 alone. This effect might have been due to losses at TP53 or at the more distal tumour-suppressor gene locus.

*erb*B-2 gene amplification was examined in the 42 cases informative for D17S5. Fifteen cases (36%) had amplification of *erb*B-2 sequences. Representative examples of tumours with *erb*B-2 gene amplification are shown in Figure 3. The amplification was 3- to 5-fold in 11 cases and >5-fold in four cases.

To assess the relative utility of LOH at D17S5 and *erb*B-2 amplification as prognostic indicators, we analysed disease-free interval with respect to these genetic events. Kaplan–Meier analysis showed that patients with LOH for D17S5 had a poor prognosis irrespective of *erb*B-2 gene amplification ($P = 0.04$). Patients with both LOH for D17S5 and *erb*B-2 amplification had the worst prognosis ($P = 0.01$) (Figure 4).

A combined analysis of the effect of LOH at D17S5 and amplification at *erb*B-2 (Table II) showed that either event increased the risk of recurrence, and the risk was the greastest for patients whose tumours had undergone both events (hazard ratio = 7.2). This effect was increased in patients whose lymph nodes were positive (hazard ratio = 13.2).

### Discussion

Our data are consistent with previous reports of two distinct regions of allele loss on chromosome 17p, one encompassing TP53 and the other more distal. In accordance with previous reports on sporadic breast cancer, the frequency of LOH observed for the telomeric region of chromosome 17p was higher than that observed for TP53 (Coles *et al.*, 1990; Sato

**Table II** Risk of recurrence according to chromosome 17 markers in 42 primary breast cancer patients

| Categories | Hazard ratio[a] Crude[b] | P value | Adjusted by nodal status[c] | P value |
|---|---|---|---|---|
| D17S5 no loss/single copy *erb*B-2 | 1.0[d] | | 1.0 | |
| D17S5 no loss/amplification *erb*B-2 | 3.7 | | 3.3 | |
| D17S5 LOH/single copy *erb*B-2 | 7.2 | | 5.0 | |
| D17S5 LOH/amplification *erb*B-2 | 6.8 | 0.029[e] | 13.2 | 0.010 |

[a]Cox proportional hazards model. [b]Hazard ratio considering the whole population, negative and positive patients for lymph node status. [c]Hazard ratio considering only patients lymph node positive. [d]Reference category, patients without any of the genetic alterations examined. [e]Patients with LOH at D17S5 and *erb*B-2 amplification *vs* patients without these genetic alterations.

**Figure 3** *erb*B-2 gene amplification in the tumours informative for D17S5. Southern and dot-blot analyses were performed as described in Materials and methods. Patient 92 shows D17S5 no loss and *erb*B-2 single copy; patient 101 shows D17S5 no loss and 3 to 5-fold *erb*B-2 amplification; patient 69 shows D17S5 allelic loss and *erb*B-2 single copy; and patient 28 shows D17S5 allelic loss and *erb*B-2 >5-fold amplification. N, normal tissue DNA; T, tumour tissue DNA.

**Figure 4** Kaplan–Meier curves for disease-free survival in primary breast cancer patients, stratified according to LOH for D17S5 and/or *erb*B-2 amplification. Cox–Mantel log-rank analysis were used to compare the D17S5 no loss/*erb*B-2 single-copy group with patients with one or both genetic events. The *P*-values for these comparisons were: D17S5 no loss/*erb*B-2 amplified, $P = 0.062$; D17S5 loss/*erb*B-2 single copy, $P = 0.04$; D17S5 loss/*erb*B-2 amplified, $P = 0.01$.

*et al.*, 1990; Andersen *et al.*, 1992). Recent studies in breast cancer (Cropp *et al.*, 1990; Andersen *et al.*, 1992) and in astrocytomas (Saxena *et al.*, 1992) using a probe for locus D17S34, which is telomeric to D17S5, have suggested that the minimal region of LOH, and thus the location of a putative tumour-suppressor gene lies between D17S5 and D17S34.

If there is another tumour-suppressor gene, distinct from TP53, on chromosome 17p, its role in breast cancer progression is unclear. Thompson *et al.* (1990) showed an association between LOH for D17S5 and low levels of oestrogen receptor, which is an indicator of poor prognosis. However, in agreement with other authors, our data showed that LOH of D17S5 is not associated with clinicopathological prognostic variables such as age, tumour size, clinical stage or steroid hormone receptor content (Cropp *et al.*, 1990; Andersen *et al.*, 1992). Nor did D17S5 LOH correlate with lymph node involvement, at present considered the most important prognostic factor in breast cancer. However, patients with LOH for D17S5, but retaining heterozygosity at TP53, had a poorer prognosis than patients who retained heterozygosity for D17S5.

Bivariate analysis of these data suggests that LOH at D17S5 is a useful prognostic indicator in breast cancer, independent of lymph node involvement. Up to one-third of patients with lymph node-negative breast cancer without adjuvant treatment relapse within 10 years (Early Breast Cancer Trialists' Collaborative Group, 1992). Thus the identification of prognostic factors, independent of the lymph node status, which can predict the course of the disease is one of the most important goals in breast cancer research. Our results suggest that LOH at loci in the telomeric region of chromosome 17p might be one such independent factor. This is consistent with studies showing an association between LOH at D17S5 and high proliferative index in breast tumours (Chen *et al.*, 1991; Merlo *et al.*, 1992).

We found no association between the occurrence of LOH for D17S5 and *erb*B-2 gene amplification. This is consistent with some reports (Børrensen *et al.*, 1990; Varley *et al.*, 1991), but not others (Sato *et al.*, 1991; Knyazev *et al.*, 1993). Bivariate analyses suggested that LOH for D17S5 and *erb*B-2 amplification are each independently associated with a poor prognosis (Table II). Further, a combination of these two genetic events and lymph node involvement provides an even stronger indication of patient prognosis. Patients with LOH for D17S5 alone or with LOH for D17S5 (but not TP53) and with *erb*B-2 amplification had a 7-fold increased risk of recurrence over patients negative for these genetic events. The risk of recurrence was increased 13-fold for lymph node-positive patients with both genetic alterations over lymph node-positive patients without these genetic changes. These data suggest that the use of nodal status combined with the analyses of genetic alterations might identify a group of patients with more aggressive disease.

We are grateful to Dr Mattias Kraus and Dr Ian J. Jacobs for kindly providing the *erb*B-2 cDNA probe and TP53 primers respectively. We thank Drs C. Eng, S. Smith and I. Jacobs for helpful discussions during the preparation of this manuscript. We appreciate the technical assistance of Sibeli Salaorini and Lidia Yamamoto. This work was supported in part by grants from the Commission of the European Communities (ERBCISTG920024), Fundação de Amparo a Pesquisa do Estado de São Paulo (91/3581-3) and Conselho Nacional de Desenvolvimento Científico e Tecnológico- CNPq/PADCT (62.427.91.4). B.A.J. Ponder is a Gibb Fellow of the CRC.

### References

ANDERSEN, T.I., GAUSTAD, A., OTTESTAD, L., FARRANTS, G.W., NESLAND, J.M., TVEIT, K.M. & BØRRESEN, A.-L. (1992). Genetic alterations of the tumor supressor gene regions 3p, 11p, 13q, 17p, and 17q in human breast carcinomas. *Genes, Chrom. Cancer*, **4**, 113–121.

BØRRENSEN, A.-L., OTTESTAD, L., GAUSTAD, A., ANDERSEN, T.I., HEIKKILA, R., JAHNSEN, T., TVEIT, K.M. & NESLAND, J.M. (1990). Amplification and protein overexpression of the neu/HER-2/c-*erb*B-2 protooncogene in human breast carcinomas: relationship to loss of gene sequences on chromosome 17, familial history and prognosis. *Br. J. Cancer*, **62**, 585–590.

BRENTANI, M.M., NAGAI, M.A., FUJIYAMA, C.T. & GOES, J.C.S. (1981). Steroid receptors in a group of Brazilian breast cancer patients. *J. Surg. Oncol.*, **18**, 431–439.

CHEN, L.-C., NEUBAUER, A., KURISU, W., WALDMAN, F.M., LJUNG, B.-M., GOODSON, W., GOLDMAN, E.S., MOORE, D., BALAZS, M., LIU, E., MAYALL, B.H. & SMITH, H.S. (1991). Loss of heterozygosity on the short arm of chromosome 17 is associated with high proliferative capacity and DNA aneuploidy in primary breast cancer. *Proc. Natl Acad. Sci. USA*, **88**, 3847–3851.

COLES, C., THOMPSON, A.M., ELDER, P.A., COHEN, B.B., MACKENZIE, I.M., CRANSTON, G., CHETTY, U., MACKEY, J., MACDONALD, M., NAKAMURA, Y., HOYHEIN, B. & STEEL, C.M. (1990). Evidence implicating at least two genes on chromosome 17p in breast carcinomas. *Lancet*, **336**, 761–763.

COLES, C., CONDIE, A., CHETTY, U., STEEL, C.M., EVANS, H.J. & PROSSER, J. (1992). p53 mutations in breast cancer. *Cancer Res.*, **52**, 5291–5298.

COX, D.R. (1972). Regression models and life-tables. *J. R. Stat. Soc.*, **34**, 187–220.

CROPP, C.S., LIDEAREAU, R., CAMPBELL, G., CHAMPENE, M.H. & CALLAHAN, R. (1990). Loss of heterozygosity on chromosome 17 and 18 in breast carcinoma: two additional regions identified. *Proc. Natl Acad. Sci. USA*, **87**, 7737–7741.

DEVILEE, P., VAN VLEIT, M., VAN SLOUN, P., DIJKSHOORN, N.K., HERMANS, J., PEARSON, P.L. & CORNELISSE, C.J. (1991). Allelotype of human breast carcinoma: a second major site for loss of heterozygosity is on chromosome 6q. *Oncogene*, **6**, 1705–1711.

EARLY BREAST CANCER TRIALISTS' COLLABORATIVE GROUP (1992). Systemic treatment of early breast cancer by hormonal, cytotoxic, or immune therapy. *Lancet*, **339**, 1–15.

EASTON, D.F., BISHOP, D.T., FORD, D., CROCKFORD, G.P. & THE BREAST CANCER LINKAGE CONSORTIUM (1993). Genetic analysis in familial breast and ovarian cancer: results from 214 families. *Am. J. Hum. Genet.*, **52**, 678–701.

FEINBERG, A.P. & VOGELSTEIN, B. (1983). A technique for radiolabeling DNA restriction endonuclease fragments to high specific activity. *Anal. Biochem.*, **132**, 6–13.

FUTREAL, P.A., BARRET, J.C. & WISEMAN, R.W. (1991). An Alu polymorphism intragenic to the TP53 gene. *Nucleic Acids Res.*, **19**, 6977.

HALL, J.M., LEE, M.K., NEWMAN, B., MORROW, J.E., ANDERSON, L.A., HUEY, B. & KING, M.-C. (1990). Linkage of early-onset familial breast cancer to chromosome 17q21. *Science*, **250**, 1684–1689.

INTERNATIONAL UNION AGAINST CANCER (1978). *Classification of Human Tumors*, 2nd edn. UICC: Geneva.

JACOBS, I.J., SMITH, S.A., WISEMAN, R.W., FUTREAL, P.A., HARRINGTON, T., OSBORNE, R.J., LEECH, V., MOLYNEUX, A., BERCHUCK, A., PONDER, B.A.J. & BAST, R.C. (1993). A deletion unit on chromosome 17q in epithelial ovarian tumors distal to the familial breast/ovarian cancer locus. *Cancer Res.*, **53**, 1218–1221.

KAPLAN, E.L. & MEIER, P. (1958). Nonparametric estimation from incomplete observations. *J. Am. Stat. Assoc.*, **53**, 457–481.

KNYAZEV, P.G., IMYANITOV, E.N., CHERNITSA, O.I. & NIKIFOROVA, I.F. (1993). Loss of heterozygosity at chromosome 17p is associated with HER-2 amplification and lack of nodal involvement in breast cancer. *Int. J. Cancer*, **53**, 11–16.

LEONE, A., MCBRIDGE, O.W., WESTON, A., WANG, M.G., ANGLARD, P., CROPP, C.S., GOEPEL, J.R., LIDEAREAU, R., CALLAHAN, R., LINEHAN, W.M., REES, R.C., HARRIS, C.C., LIOTTA, L.A. & STEEG, P.S. (1991). Somatic allelic deletions of NM23 in human cancer. *Cancer Res.*, **51**, 2490–2493.

MACKAY, J., STEEL, C.M., ELDER, P.A., FORREST, A.P.M. & EVANS, H.J. (1988). Allele loss on short arm of chromosome 17 in breast cancer. *Lancet*, **ii**, 1384–1385.

MALKIN, D., LI, F.P., STRONG, L.C., FRAUMENI, J.F., NELSON, C.E., KIM, D.H., KASSEL, J., GRYKA, M.A., OSCHOP, J.F.Z., TAINSKY, M.A. & FRIEND, S.H. (1990). Germ line p53 mutations in a familial syndrome of breast cancer, sarcomas and other neoplasms. *Science*, **250**, 1233–1238.

MARQUES, L.A., FRANCO, E.L., TORLONI, H., BRENTANI, M.M., SILVA-NETO, J.B. & BRENTANI, R.R. (1990). Independent prognostic value of laminin receptor expression in breast cancer survival. *Cancer Res.*, **50**, 1479–1483.

MAZARS, R., SPINARDI, L., BENCHEIKH, M., SIMONY-LAFONTAINE, J., JEANTEUR, P. & THEILLET, C. (1992). p53 mutations occur in aggressive breast cancer. *Cancer Res.*, **52**, 3918–3923.

MERLO, G.R., VENESIO, T., BERNARDI, A., CANALE, L., GAGLIA, P., LAURO, D., CAPPA, A.P.M., CALLAHAN, R. & LISCIA, D.S. (1992). Loss of heterozygosity on chromosome 17p13 in breast carcinomas identifies tumors with high proliferation index. *Am. J. Pathol.*, **140**, 215–223.

MULLIGAN, L.M., MATLASHEWSKI, G.J., SCRABLE, H.J. & CAVENEE, W.K. (1990). Mechanisms of p53 loss in human sarcomas. *Proc. Natl Acad. Sci. USA.*, **87**, 5863–5867.

NAKAMURA, Y., BALLARD, L., LEPPERT, M., O'CONNELL, P., LATHROP, G.M., LALOUEL, J.M. & WHITE, R. (1988). Isolation and mapping of a polymorphic DNA sequence pYNZ22 on chromosome 17p (D17S30). *Nucleic Acids Res.*, **16**, 5707.

SATO, T., TANIGAMI, A., YAMAKWA, K., AKIYAMA, F., KASUMI, F., SAKAMOTO, G. & NAKAMURA, Y. (1990). Allelotype of breast cancer: Cumulative allele losses promote tumor progression in primary breast cancer. *Cancer Res.*, **50**, 7184–7189.

SATO, T., AKIYAMA, F., SAKAMOTO, G., KASUMI, F. & NAKAMURA, Y. (1991). Accumulation of genetic alterations and progression of primary breast cancer. *Cancer Res.*, **51**, 5794–5799.

SAXENA, A., CLARK, W.C., ROBERTSON, J.T., IKEJIRI, B., OLDFIELD, E.H. & ALI, I.U. (1992). Evidence for the involvement of a potential second tumor suppressor gene on chromosome 17 distinct from p53 in malignant astrocytomas. *Cancer Res.*, **52**, 6716–6721.

TAKITA, K.-I., SATO, T., MIYAGE, M., WATATANI, M., AKIYAMA, F., SAKAMOTO, G., KASUMI, F., ABE, R. & NAKAMURA, Y. (1992). Correlation of loss of alleles on the short arms of chromosomes 11 and 17 with metastasis of primary breast cancer to lymph nodes. *Cancer Res.*, **52**, 3914–3917.

THOMPSON, A.M., STEEL, C.M., CHETTY, U., HAWKINS, R.A., MILLER, W.R., CARTER, D.C., FORREST, A.P.M. & EVANS, H.J. (1990). p53 gene mRNA expression and chromosome 17p allele loss in breast cancer. *Br. J. Cancer*, **61**, 74–78.

VARLEY, J.M., BRAMMAR, W.J., LANE, D.P., SWALLOW, J.E., DOLAN, C. & WALKER, R.A. (1991). Loss of chromosome 17p13 sequences and mutations of p53 in human breast carcinomas. *Oncogene*, **6**, 413–421.

WORLD HEALTH ORGANIZATION (1981). *Histologic Typing of Breast Tumors*, 2nd edn. WHO: Geneva.